# Artificial intelligence addiction among university students in China: risk stratification, consequences, and exercise-based intervention

**DOI:** 10.3389/fpsyg.2026.1779639

**Published:** 2026-06-25

**Authors:** Gengdan Hu, Yinan Zhou, Chuyan Zhang, Yipeng Sha

**Affiliations:** 1 Clinical Research Center for Mental Disorders, Shanghai Pudong New Area Mental Health Center, School of Medicine, Tongji University, Shanghai, China; 2 Department of Psychology, School of Humanities, Tongji University, Shanghai, China; 3 Department of Physical Education (International College of Football), Tongji University, Shanghai, China; 4 School of Ocean and Earth Science, Tongji University, Shanghai, China; 5 Research Center for the Theory of Socialism with Chinese Characteristics, Tongji University, Shanghai, China

**Keywords:** artificial intelligence addiction, behavioral addiction, mental health, physical exercise intervention, real-life consequences, university students

## Abstract

With the deep integration of artificial intelligence into daily life, AI-addiction-like tendencies have emerged as a potential form of behavioral dependency among university students. Focusing on high-frequency AI users on campus, this study examined risk stratification of problematic AI use, real-life impacts, and the preliminary pre-post improvements following exercise-based interventions. A cross-sectional survey assessed AI usage patterns and functional impacts, identifying a substantial proportion of students with at least mild risk of problematic AI use. Problematic AI use was associated with reduced academic performance, social engagement, physical health, personal interests, and emotional well-being. A six-month structured exercise intervention delivered to at-risk student was associated with reductions in problematic AI use risk and improvements in daily functioning. These findings provide preliminary support for conceptualizing AI addiction as a multidimensional behavioral dependency potentially distinguishable from traditional Internet addiction and suggest that structured exercise may hold potential as a non-pharmacological approach for mitigating maladaptive AI use in university settings.

## 1 Introduction

Since the beginning of the 21^st^ century, the rapid development and deep penetration of Internet technologies have brought the phenomenon of Internet addiction to the forefront of psychology, education, and sociology. As early as the 1990s, it was conceptualized as a behavioral addiction, with diagnostic criteria and theoretical frameworks gradually established to support empirical research (Young, 1998). Later reviews underscored its high prevalence among adolescents and confirmed its core symptoms—tolerance, withdrawal, diminished control over use, and functional impairment—align with the broader paradigm of behavioral addictions (Kuss and Griffiths, 2017).

These perspectives have gained broad recognition within the academic community. The American Psychiatric Association (APA) included “Internet Gaming Disorder” in the *Diagnostic and Statistical Manual of Mental Disorders (5*^*th*^ *ed, DSM-5)* as a condition

warranting further study, marking the formal inclusion of Internet-related behavioral issues within the scope of mental health research. Building on this foundation, prior research has explored behavioral interventions targeting problematic digital use. In the Chinese context, studies drawing on innate psychological needs theory argued that Internet addiction is associated with deficits in belongingness, competence, and autonomy in real-life environments. Physical exercise, as a structured and engaging activity, may help restore these needs and has been associated with reductions in addictive symptoms (Hu and Zhang, 2016). AI use may similarly reflect compensation for unmet psychological needs; thus, exercise-based interventions may support behavioral regulation in AI-related addictive behaviors.

However, the rise of the Artificial Intelligence Era has fundamentally reshaped the mechanisms of digital engagement. AI has shifted from a supporting tool to the core driver of platform operations. Its real-time data processing, adaptive learning, and personalized modeling capabilities enable precise need detection, dynamic content delivery, and continuous behavioral reinforcement. While these systems enhance user experience, they also intensify psychological dependence, giving rise to a more complex phenomenon: AI addiction.

Compared with traditional Internet technologies, AI systems introduce distinct addictive dynamics. Their continuously optimized feedback loops create highly individualized and instantly gratifying interactions, gradually reinforcing excessive use. Through natural language processing and affective modeling, AI further provides interpersonal responses that meet emotional and relational needs—an affordance far beyond earlier online environments (Al-Obaydi and Pikhart, 2025). This simulated empathy may promote deepened reliance and even emotional attachment. Moreover, AI products are often framed as tools for efficiency and enhancement, obscuring their potentially addictive design features and complicating both ethical and technical identification. These differences suggest that AI addiction involves distinct interaction mechanisms and should be examined beyond conventional Internet addiction frameworks.

The risks are substantial: AI addiction may impair cognition, undermine real-world social functioning, diminish learning efficiency, and compromise mental health. These challenges indicate that AI addiction should not be treated as merely a variant of Internet addiction but as an emerging domain driven by unique technological architecture and psychosocial mechanisms. Yet, existing theoretical frameworks remain fragmented, with inconsistencies across conceptual definitions, limited consensus on measurement approaches, and still-developing etiological models and intervention strategies.

To address this gap, the present study investigates potentially addictive AI use among university students and its associations with perceived negative impacts on real-life functioning. University students are characterized by high exposure to AI technologies, making them a particularly suitable population for investigating emerging patterns of such use.

Grounded in behavioral addiction framework, it explores cognitive, emotional, and social correlates of AI-dependent use patterns and assesses physical exercise as a potential non-pharmacological intervention. This approach is informed by prior research suggesting that maladaptive digital engagement may be linked to unmet psychological needs, which can be partially restored through structured physical activity, thereby supporting behavioral regulation.

Rather than establishing a formal diagnostic category, the study seeks to generate preliminary empirical evidence resembling addictive patterns within a non-clinical university sample to inform ongoing conceptual clarification and future longitudinal inquiry. Through this dual focus on impact assessment and intervention, the study aims to contribute to a more nuanced understanding of potentially addictive AI use.

## 2 Literature review

AI-driven interactions differ substantially from traditional digital technologies in both design logic and psychological impact, leading to new forms of dependence that extend beyond conventional Internet addiction models. This section synthesizes existing scholarships by reviewing conceptual definitions, characteristic features, and underlying mechanisms of AI addiction, thereby providing a coherent theoretical framework for the empirical and intervention analyses that follow.

### 2.1 Definition

Although “AI addiction” has not yet been formally incorporated into diagnostic frameworks such as the *DSM-5*, it is widely regarded as an emerging subtype of behavioral addiction. Consistent with classical models of problematic Internet use, AI addiction involves compulsive engagement, diminished control, psychological withdrawal, and functional impairment (Davis, 2001; Kalivas and Volkow, 2005; Prochaska et al., 1992).

In essence, AI addiction could be viewed as the extension and evolution of Internet addiction in an era where interactive agents become the primary objects of dependence. Whereas, traditional Internet addiction is often activity-specific (e.g., gaming, social networking) (Van Rooij and Prause, 2014), AI addiction centers on interactions with systems capable of generating personalized, adaptive, and anthropomorphic responses.

This perspective aligns with a long-standing scholarly debate on whether Internet addiction reflects dependence on the Internet as a general medium or on specific online activities with inherent addictive potential.

The Internet, as a heterogeneous technological environment encompassing entertainment, social interaction, consumption and information retrieval, makes the broad notion of “Internet addiction” insufficient to explain the diverse motivational and clinical profiles of different problematic behaviors. Consequently, research has shifted activity-based classifications, such as Internet Gaming Disorder in the *DSM-5* appendix and growing study on social media and online shopping addictions.

Similar heterogeneity characterizes AI addiction. Artificial intelligence is not a singular technology but a constellation of interaction models, including generative AI, conversational AI, and algorithmically curated content systems. These systems differ substantially in interactivity, motivational affordances, and risk patterns, giving rise to distinct addictive subtypes.

Existing evidence highlights three primary forms: (a) Conversational AI addiction or Problematic Use of Conversational AI (PUCAI), driven by loneliness, social anxiety, and anthropomorphic perceptions (Hu et al., 2023); (b) Generative AI addiction, shaped by immersive flow experiences and immediate creative feedback (Zhou and Zhang, 2024); and (c) Algorithmic addiction, rooted in personalized recommendation algorithms and “dark patterns” (e.g., infinite scrolling, auto-play, ambiguous exit buttons) that induce compulsive engagement (Nie, 2025). These subtypes illustrate that AI Addiction is not a unitary construct but rather a multidimensional spectrum of behavioral dependencies shaped by diverse human-AI interaction mechanisms.

This heterogeneity warrants a correspondingly diverse set of tailored measurement instruments for substantive study. However, there is currently a lack of widely established diagnostic criteria or formal clinical instruments for AI addiction. Cross-culturally validated measurement scales also remain limited, and existing tools may not fully encompass heterogeneous forms of AI engagement. Consequently, rather than adopting a narrow subtype-specific definition that may exclude emerging or hybrid forms of AI interaction, this study employs a broad conceptualization to accommodate the diversity of AI engagement.

Within this framework, AI addiction is defined as compulsive use and uncontrollable reliance on AI systems driven by emotional gratification, social substitution, or cognitive rewards, resulting in marked impairment in academic functioning, interpersonal relationships, and mental health. This definition preserves the core features of behavioral addictions while foregrounding AI-specific characteristics such as anthropomorphic design, rapid feedback loops, and personalized algorithmic reinforcement that accelerate the formation and maintenance of addictive behaviors.

The construct is intended to distinguish between high frequency but adaptive use and patterns that are associated with psychological distress or impairment in daily functioning. Therefore, the term “risk level” is adopted throughout this study to avoid implications and to emphasize the exploratory nature of the construct.

### 2.2 Characteristic features

Considering that AI Addiction could be viewed as an extension and evolution of Internet Addiction within the context of artificial intelligence technologies, understanding its core feature requires reference to the established characteristics of Internet addiction. Prior research shows that Internet addiction manifests as excessive and prolonged online engagement, disrupted daily routines, physical fatigue, and declining academic or work performance. Individuals often experience difficulty withdrawing from online environments and require increasing usage to achieve similar levels of satisfaction (Young, 2004). These features form the conceptual foundation for analyzing AI-specific addictive patterns.

Building upon this foundation, four prominent characteristics of AI addiction could be identified:

1. **Difficulty in Controlling Use**: Loss of control is a central criterion of behavioral addiction. Individuals with AI addiction struggle to regulate their time spent interacting with AI tools, frequently interrupting study, work, or daily activities to engage with AI systems. Usage persists despite the negative consequences, mirroring the impaired control commonly observed in Internet addiction.
2. **Emotional Dependency**: The immediacy and predictability of AI-generated feedback amplify psychological dependence (Brand et al., 2019). Many users rely on AI systems for emotional soothing or regulation, and when access is restricted, they may experience symptoms resembling withdrawal observed in Internet Gaming Disorder such as restlessness, irritability, or mood instability.
3. **Cognitive Offloading**: AI addiction often involves habitual delegation of cognitive tasks (e.g., writing, problem-solving, or decision-making) to AI systems. Persistent reliance on AI-generated content reduces opportunities for independent thinking and may undermine critical thinking and problem-solving skills (Sparrow et al., 2011; Wilmer et al., 2017). This tendency becomes particularly pronounced with generative AI, which significantly lowers the effort required for idea generation or creativity.
4. **Social Dependency**: Some individuals increasingly depend on AI systems for social support or validation. As emotional expression and interpersonal needs become displaced onto AI, real-world social interactions may diminish, potentially resulting in social withdrawal or impaired social functioning (Zhou and Zhang, 2024).

The four characteristics are not proposed as a newly developed construct. Rather, they are derived from and integrated across established behavioral addiction theories, widely used Internet addiction assessment, and emerging empirical studies on AI use. Specifically, control difficulties and emotional dependence are adapted from behavioral addiction frameworks, while cognitive offloading and social dependence extend these models by reflecting AI interaction patterns identified in recent literature.

In summary, while AI addiction inherits core symptoms from traditional Internet addiction, its risk patterns are amplified by unique human-AI interaction mechanisms. These features provide the conceptual groundwork for understanding the psychological mechanisms underlying AI addiction and for informing targeted intervention strategies.

### 2.3 Mechanism

Emerging studies suggest that AI addiction may arise from an interplay of neurobiological, cognitive-behavioral, design-related, and psychosocial mechanisms.

Drawing from developed models of Internet addiction, researchers highlight that excessive engagement with highly stimulating digital environments can disrupt the balance between reward processing and executive control. Functional and structural alterations in reward circuits, coupled with diminished prefrontal regulatory capacity, may increase sensitivity to immediate feedback and reinforce compulsive interaction patterns (Chen et al., 2023).

From a behavioral learning perspective, conversational AI systems such as ChatGPT provide highly personalized, immediate, and often unpredictable responses. This pattern resembles variable-ratio reinforcement, long recognized as a potent driver of compulsive engagement in contexts such as gambling and social media. Each interaction carries the possibility of informational gain, emotional validation, or problem resolution, which strengthens the motivation to re-engage and sustains habitual use (Yankouskaya and Liebherr, 2025).

At the technological and design level, persuasive design strategies amplify these reinforcement mechanisms. Features including real-time responsiveness, tailored prompts, and interaction continuity reduce friction costs and maintain attentional capture. Reviews of persuasive technologies emphasize that, while such strategies can support beneficial behavior change, they also risk fostering high-frequency engagement that gradually undermines self-monitoring and volitional control (Idrees et al., 2024).

Psychosocial processes further contribute to the formation of dependence-like dynamics. Conversational AI offers responsive, “non-judgmental interaction”, which may be particularly appealing to individuals experiencing loneliness, anxiety, or unmet emotional needs. This socio-emotional substitution can weaken motivation for offline social engagement and foster emotional reliance on AI companions. Empirical findings suggest that compulsive AI use may, in turn, exacerbate anxiety, burnout, and sleep disturbances, creating a feedback loop that reinforces problematic engagement (Duong et al., 2024).

Finally, scholars emphasize that “AI addiction” remains a developing construct. Existing models largely extend frameworks from behavioral addictions rather than identify entirely novel mechanisms. Ongoing debates call for clearer conceptual boundaries, longitudinal evidence, and neurobiological validation to distinguish high involvement from clinically meaningful addictive patterns.

### 2.4 Intervention

Recent research suggests that structured physical activity can mitigate behavioral addictions, including AI-related maladaptive use, through integrated biological, psychological, and social mechanisms. The exercise substitution model posits that systematic engagement in sports can satisfy intrinsic psychological needs such as autonomy, competence, and relatedness, thereby replacing compulsive digital behaviors (Hu and Zhang, 2016). Neurobiologically, moderate-intensity aerobic exercise enhances dopaminergic signaling, BDNF expression, and endocannabinoid activity, promoting neural plasticity, stabilizing reward circuits, and improving executive control, which together reduce impulsivity and reward dependence.

Psychologically, exercise provides structured routines and goal-directed activities, enhancing self-efficacy, emotional regulation, and cognitive control. Participation in group sports fosters social engagement, cooperation, and conflict resolution, supporting recovery from maladaptive online behaviors (Zhou and Zhang, 2024). From a social perspective, personalized interventions that consider individual preferences, addiction severity, and social context optimize engagement and adherence. Socially embedded practices, including peer interaction and community support, further reinforce healthy habits and reduce reliance on algorithmic reward loops.

The integrated framework emphasizes multi-dimensional evaluation, combining behavioral outcomes, psychological functioning, and social adaptability. Sustained intervention effects require habit maintenance and supportive family-school-community systems to provide guidance, opportunities, and conductive environments. Empirical evidence demonstrates that exercise interventions can broadly apply to behavioral addictions, suggesting potential effectiveness for addressing AI-related maladaptive use and laying a foundation for personalized, theoretically grounded intervention strategies (Brown et al., 2009).

## 3 Method

### 3.1 Participants and procedure

This study targeted high-frequency users of AI technology, with a particular focus on Chinese university students. The research was conducted in two sequential phases: an initial cross-sectional survey designed to assess AI addiction risk and its real-life impact, followed by an exercise-based intervention aimed at examining the potential for behavioral risk reduction.

In the first phase, data were collected through a large-scale online survey distributed via *Wenjuanxing*, a professional questionnaire platform. The survey was administered from July 27 to 29, 2025. Prior to participation, all respondents were provided with detailed information regarding the study objectives, voluntary nature of participation, data confidentiality, and privacy protection measures. Electronic informed consent was obtained before participation.

In total, 246 questionnaires were distributed, of which 241 were deemed valid after excluding incomplete or logically inconsistent responses, yielding a response rate of 97.9%. The final sample consisted of 121 males and 120 females. The survey data were used to examine AI addiction risk stratification and its associated perceived negative impact on daily functioning.

Based on the total scores of the “AI Usage Status” module, participants were classified into four AI addiction risk levels: normal use, mild risk, moderate risk, and severe risk. Individuals identified as being at mild or moderate risk of AI addiction were invited to participate in a subsequent exercise intervention to further evaluate the feasibility of exercise-based intervention. Participation in the intervention phase was voluntary.

The exercise intervention was conducted over a six-month period, from 1 August 2025 to 31 January 2026. 32 students consented to participate and completed the intervention protocol, reporting their exercise activities throughout the program. Self-reported exercise data were cross validated with objective exercise monitoring records derived from large-scale physical activity tracking data. Based on these objective verifications, two moderate-risk participants were excluded from the final analysis due to insufficient exercise intensity. The final analytic sample therefore comprised 30 participants (24 mild-risk and 6 moderate-risk) for outcome evaluation.

Pre-intervention AI usage risk was assessed using the “AI Usage Status” module, and the same measure was re-administered immediately after the completion of the program to evaluate changes in AI-related maladaptive use.

### 3.2 Instruments

#### 3.2.1 Questionnaire development

Research on problematic digital engagement has long relied on psychometrically validated instruments to ensure reliable and interpretable measurement. In the domain of Internet-related behaviors, tools such as the *Internet Addiction Test* (IAT) have been extensively examined for internal consistency and construct validity. In a large sample of Chinese adolescents, the IAT demonstrated strong internal consistency (Cronbach's α = 0.93) and a stable factor structure supported by confirmatory factor analysis, indicating robust psychometric properties in capturing dimensions of problematic Internet use (Lai et al., 2013).

Moreover, the *Revised Chen Internet Addiction Scale* (CIAS-R) has shown high reliability and construct validity, with factor analytic evidence revealing meaningful subdimensions of Internet use problems and strong inter-item correlations in adolescent samples (Mak et al., 2014). Systematic reviews and meta-analyses further confirm that the IAT generally exhibits acceptable internal consistency and convergent validity across diverse populations, affirming its utility for research on maladaptive online behaviors (Moon et al., 2018).

Drawing on psychometrically grounded assessment tools, a preliminary instrument was developed to assess problematic AI use risk among Chinese university students. *The Survey on the Usage of AI Tools and Services* incorporated conceptual dimensions from the IAT and CIAS-R, targeting maladaptive digital engagement such as compulsive use, emotional dependence, and interference with daily functioning.

The “AI Usage Status” module reflects the IAT's focus on difficulty in usage control and preoccupation with online activities, whereas cognitive offloading and social dependence were derived from the CIAS-R subscales on cognitive preoccupation and social problem. Additionally, the Perceived Negative Impact module was developed based on the CIAS-R functional impairment dimension, assessing perceived impacts across five domains: learning/work efficiency, social interaction, physical health, hobbies, and emotional wellbeing.

#### 3.2.2 Questionnaire content

The questionnaire consisted of five modules: 1. Basic Information: Collected demographic data including age, gender, status, and educational background.

2. AI Usage Status: Measured four dimensions four dimensions: “difficulty in usage control”, “emotional dependence”, “cognitive offloading”, and “social dependence”. Prior to completing this section, respondents were provided with a standardized definition of “AI tools and services”, which explicitly includes conversational AI, generative AI, and algorithmically curated systems. Respondents were instructed to evaluate their overall AI usage experience to the survey items, thereby ensuring that the measurement captured a unified construct of AI engagement across heterogeneous contexts.

Items were rated on a 5-point Likert scale (1 = Never, 5 = Always) with higher scores indicating greater risk of problematic AI use. All items were positively keyed toward problematic AI use; therefore, no reverse scoring procedures were required.

Responses were summed to yield a total score ranging from 16 to 80, which was used to classify problematic AI use risk: Normal use (16–39), Mild risk (40–55), Moderate risk (56–71), and Severe risk (72–80). Scores ≥ 40 were considered indicative of potential problematic AI use, as this threshold corresponded to the empirically identified transition region in the score distribution and was supported by cluster-based classification analysis.

Cut-off values were set for exploratory risk stratification rather than diagnostic purposes, guided by score distributions and conceptual alignment with behavioral addiction severity. These thresholds indicate relative risk levels rather than clinical diagnoses.

3. Real-life Impact: Assessed the negative effects of AI tool usage across five dimensions: “learning/work efficiency”, “real-world social interaction”, “physical health”, “hobbies”, and “emotional state”. Responses were collected on a 5-point Likert scale was used (1 = Strongly Disagree, 5 = Strongly Agree), with higher scores indicating greater perceived negative impact.

4. Physical Exercises: Comprised single-choice and multiple-choice questions to evaluate participants' exercise habits and attitudes toward sports. An open-ended question—“What support do you hope schools or communities will provide to promote sports? (e.g., free venues, group courses)”—captured qualitative feedback.

Physical exercise intensity was operationally defined based on both the frequency and duration of participants' exercise sessions. Intensity levels were categorized as follows: participants reporting no exercise were classified as Low intensity. For exercises performed monthly, sessions lasting 10–30 min or 30 min were classified as Low, while sessions of 1–2 h were classified as Medium; durations outside these ranges were coded as Unclear. For exercises performed weekly, sessions of 10–30 min or 30 min were classified as Medium, whereas sessions of 1–2 h were classified as High; durations beyond these ranges were coded as Unclear.

Participants assigned to the “Unclear” group were those whose responses showed inconsistencies between reported exercise frequency and duration, or whose responses did not allow for a clear classification of exercise intensity. Only participants with internally consistent and logically corresponding frequency-duration patterns were classified into low, medium, or high intensity groups.

This operationalization allowed for standardized assessment of physical activity intensity across participants and ensured consistency in subsequent analyses of intervention effects.

5. Intervention Effect Perception: Included a matrix scale using a 5-point Likert scale to assess participants' perception of the corrective effects of physical activity on AI addiction. Multiple-choice items further explored opinions on sports-based intervention strategies.

#### 3.2.3 Questionnaire reliability and validity

The psychometric properties of the questionnaire were evaluated using the total survey sample *(N* = *241)*.

Internal consistency reliability of the questionnaire was excellent, with a Cronbach's α of 0.91. The three main modules assessing problematic AI use, real-life impact and perceived effects of exercise intervention demonstrated very good reliability, α ranging from 0.89 to 0.94. Specifically, the “AI Usage Status” module exhibited a Cronbach's α of 0.93, with its four subdimensions showing excellent reliability: difficulty in usage control (α = 0.92), emotional dependence (α = 0.90), cognitive offloading (α = 0.93), and social dependence (α = 0.90).

Content validity was first supported through expert review. Prior to formal data collection, a pilot study (*n* = *24)* was conducted to assess item clarity, semantic precision, and logical flow. Minor wording revisions were implemented based on participant feedback to enhance clarity and logical consistency prior to full deployment.

### 3.3 Exercise intervention

The exercise intervention constituted the second phase of the study and was designed as a targeted behavioral strategy for participants identified as being at AI addiction risk. Drawing upon the “Instinct-Activated Sports Intervention” framework (Hu and Zhang, 2016), the program aimed to examine whether structured and intrinsically motivated physical activity could reduce AI-related maladaptive use over time.

Participants were guided to develop intrinsic interest in sports, master at least one sport skill, and establish initial habits of autonomous exercise. They engaged in self-selected sports activities (e.g., basketball, badminton, swimming, aerobics, street dance) three times per week for 90 min per session. Weekend sessions were held off-campus under structured coaching, emphasizing moderate-intensity aerobic exercise while respecting individual differences. Participants continued regular school physical education classes. Coaches integrated intervention objectives into training sessions and provided progress feedback to ensure intervention fidelity.

Exercise participation was monitored using a multi-source verification approach, combining self-reported data and institutional physical activity records. Subjective self-reported data on exercise frequency, duration, and perceived intensity were collected through questionnaire measures. Objective behavioral data were retrieved from the institutional physical activity database, which records each exercise session through campus-based tracking systems, including date, time, and location. For aerobic activities such as running, the system further captures total duration, distance, and average pace, thereby ensuring data authenticity. The integration of self-reported and institutional data enabled cross-validation of exercise adherence and strengthened the methodological rigor of the intervention evaluation.

Two participants were excluded from subsequent analyses due to substantial discrepancies between their self-reported exercise data and objectively recorded physical activity, which compromised data validity for the intervention analysis.

### 3.4 Data analysis

Statistical analyses were conducted using SPSS 26.0.

To examine the psychometric properties of the “AI Usage Status” module, con-firmatory factor analysis (CFA) was conducted using the full sample data to assess the adequacy of the proposed factor structure. Descriptive statistics were also generated to summarize demographic characteristics and scale distributions. All analyses reported in Section 4.1 were conducted using the formal survey sample.

To establish a data-driven boundary between normative and at-risk AI users, total scores were subjected to *K*-means clustering with the number of clusters fixed at two. The midpoint between the converged cluster centers was calculated to derive an empirical classification threshold. Agreement between this empirical boundary and the theoretically predefined cut-off (score ≥ 40) was assessed using a chi-square test.

For the intervention phase, paired-samples *t* tests were conducted to evaluate pre-post differences in AI addiction risk scores among participants who completed the six-month exercise program.

## 4 Results

### 4.1 Empirical validation of the cut-off

**Figure 1:**
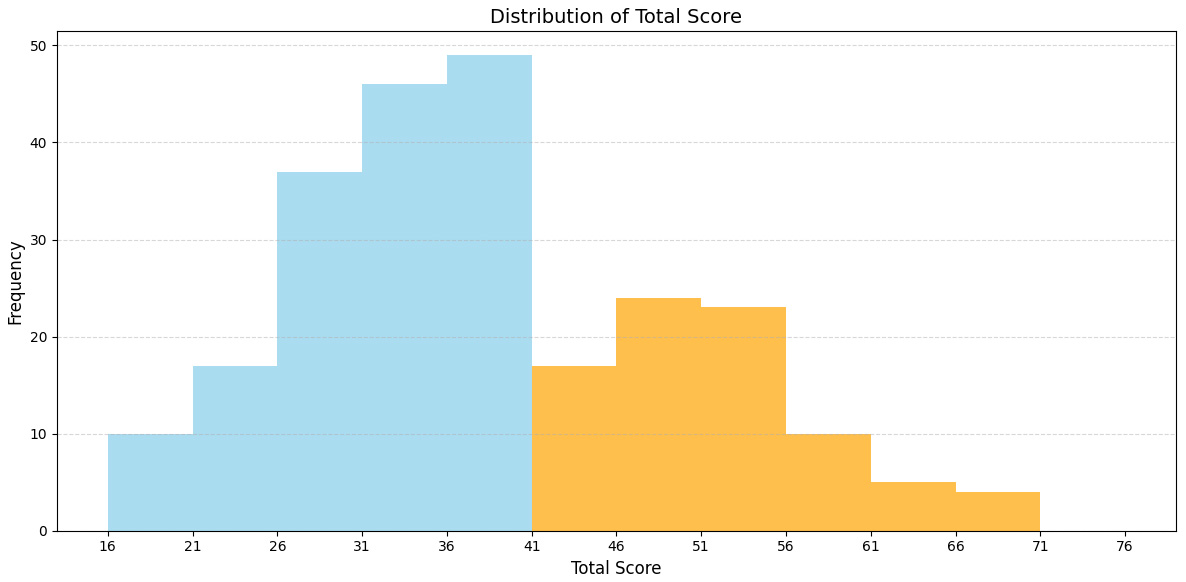
Distribution of total score.

**Figure 2:**
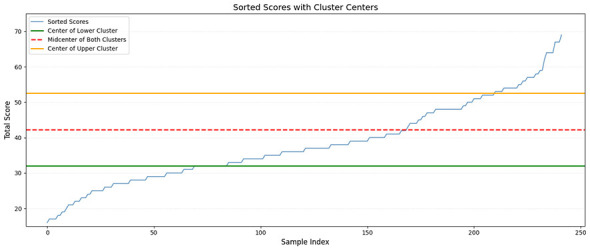
Sorted scores with cluster centers.

Total scores on the “AI Usage Status” module (Q5–Q8) among 241 respondents ranged from 16 to 80 (M = 38.05, SD = 11.53). As shown in Figure 1, the score distribution exhibited a transitional concentration zone approximately between 30 and 45, suggesting a potential boundary between normative and elevated use. Building on this distributional pattern, K-means cluster analysis (Figure 2) identified two cluster centers at 31.83 and 52.64, yielding a midpoint of 42.24. This empirically derived separation closely approximated the proposed threshold of 40, differing by only 2.24 points.

To examine the classification consistency between the empirically derived midpoint and the proposed cut-off, a chi-square test was conducted to assess whether the two criteria produced significantly different group assignments. The result indicated no significant difference, χ^2^ = 2.166, *p* = 0.141, supporting the use of 40 as a valid criterion for distinguishing at-risk users.

The findings suggest that the “AI Usage Status” scale reliably measures the four intended dimensions in a university student sample, and that the established cut-off score of 40 provides a valid criterion for distinguishing between normative and at-risk AI users. The scale could therefore be used for exploratory identifying individuals at higher risk of problematic AI use in subsequent intervention studies.

### 4.2 Addiction risk stratification

Among the 241 valid participants (121 males and 120 females), 91 individuals exhibited varying degrees of AI dependence risk, accounting for 37.76% of the total sample.

Specifically, 71 respondents showed a mild risk of AI addiction (36 males, 35 females; 29.46%), while 20 respondents exhibited a moderate risk (10 males, 10 females; 8.30%). No respondents exhibited a severe risk of AI addiction. Contingency table analysis indicated no significant gender differences in addiction status (χ^2^ = 0.001, *p* = 1.00), suggesting that tendencies and levels of AI addiction risk were comparable across male and female participants.

Drawing upon these empirical results, over one-third of university students show at least mild AI-related maladaptive use, highlighting the relevance and urgency of investigating AI addiction. The substantial proportion of students at risk underscores the necessity for targeted interventions and supports the rationale for subsequent exercise-based intervention studies to mitigate potential functional consequences associated with high-frequency AI usage.

### 4.3 Perceived negative impacts

As shown in Table 1, respondents with different levels of AI addiction risk exhibited varying degrees of perceived negative impact across core domains. At-risk AI users reported significantly higher scores than normative users across all five measured domains.

**Table 1:** Average impact scores of different AI addiction risk levels on core domains.

AI addiction risk level	Learning and work efficiency (SD)	Real-world social interaction (SD)	Physical health (SD)	Hobbies and interests (SD)	Emotional state (SD)
Normal usage	7.71 (2.45)	5.03 (2.13)	6.01 (2.63)	5.65 (2.31)	5.60 (2.02)
Mild addiction risk	9.54	6.65	9.18	9.49	8.97
Moderate addiction risk	11.60	8.95	9.95	9.90	10.35
*Avg. of at-risk group*	9.99 (2.37)	7.15 (3.11)	9.35 (3.01)	9.58 (2.88)	9.27 (2.89)
*Overall average*	8.57	5.83	7.27	7.13	6.99

The full score for each aspect is 15 points. The “Avg. of At-risk Group” is the mean of the Mild and Moderate Addiction groups. The “Overall Average” includes all groups (Normal, Mild, and Moderate).

In learning and work efficiency, at-risk users had higher scores (M = 9.99, SD = 2.37) than normative users (M = 7.71, SD = 2.45), reflecting noticeable reductions in academic and occupational performance. In social interaction, at-risk users scored higher (M = 7.15, SD = 3.11) than normative users (M = 5.03, SD = 2.13), indicating greater variability in real-world social engagement. Physical health scores were also higher among at-risk users (M = 9.35, SD = 3.01) compared to normative users (M = 6.01, SD = 2.63), demonstrating measurable negative consequences. Hobbies and personal motivation were similarly affected, with at-risk users reporting higher scores (M = 9.58, SD = 2.88) than normative users (M = 5.65, SD = 2.31). Emotional wellbeing was also impacted, as at-risk users averaged higher scores (M = 9.27, SD = 2.89) than normative users (M = 5.60, SD = 2.02), reflecting greater mood fluctuations and elevated stress.

Collectively, the *t*-test results demonstrate that at-risk AI users experience significant impacts across academic/work performance, social interaction, physical health, leisure activities, and emotional wellbeing. Effect sizes were large (Cohen's *d* = 0.83–1.55), indicating that these differences are not only statistically significant but also practically meaningful. Problematic AI use appears to exert widespread consequences on university students' daily functioning, highlighting the importance of implementing targeted intervention strategies.

### 4.4 Exercise patterns

Table 2 suggests a tendency for exercise intensity to vary across levels of AI addiction risk. Participants with normal AI use more frequently reported medium-intensity exercise (49.0%), whereas low-intensity activity was more common among mild-risk (49.0%) and moderate-risk individuals (57.1%). Engagement in higher-intensity exercise appeared less frequently in the at-risk groups.

**Table 2:** Association between AI addiction risk and physical exercise intensity.

Addiction risk level	Physical exercise intensity				Total
	Low	Medium	High	Unclear	
Normal usage	34(33.33%)	50(49.02%)	18(17.65%)	48	150
Mild addiction risk	24(48.98%)	17(34.69%)	8(16.33%)	22	71
Moderate addiction risk	8(57.14%)	3(21.43%)	3(21.43%)	6	20

The total sample size: *N* = 241. Data are presented as *n* (%), with percentages calculated within each addiction risk level (Normal, Mild and Moderate), excluding the “Unclear” group.

Overall, the pattern indicates that elevated AI addiction risk may be associated with lower levels of moderate-to-high intensity physical activity, providing a basis for exploring the potential benefits of structured exercise programs as a supportive approach to promote healthier AI use and daily functioning.

### 4.5 Intervention outcomes

This phase evaluated the effects of the six-month structured exercise intervention on AI addiction severity and real-life functioning among at-risk participants.

Following the exercise intervention, the 30 participants initially classified as at-risk showed marked improvement. The addiction risk distribution of participants before and after the intervention is presented in Table 3. All six moderate-risk participants reduced their risk level, with five reclassified as mild-risk and one achieving normal use. All the 24 mild-risk participants returned to normal range. Overall, 25 of 30 participants (83.3%) restored normal AI use at post-test.

**Table 3:** Distribution of AI addiction risk before and after intervention.

Addiction risk level	Pre-intervention	Post-intervention
Normal usage	0	25
Mild addiction risk	24	5
Moderate addiction risk	6	0

As shown in Table 4, AI addiction scores decreased from the pre-intervention (M = 48.87, SD = 7.34) to post-intervention (M = 34.50, SD = 6.28). A paired-samples *t*-test indicated a significant difference (*t* = −21.54, *p* < 0.001).

**Table 4:** Score changes in AI usage status and real-life functioning after intervention.

Measure	AI usage status total	Learning and work efficiency	Real-world social interaction	Physical health	Hobbies and interests	Emotional state
Pre-intervention mean (SD)	48.87 (7.34)	9.57 (1.45)	7.63 (2.67)	8.20 (2.38)	8.45 (2.54)	8.27 (2.42)
Post-intervention mean (SD)	34.50 (6.28)	6.73 (1.28)	5.27 (1.39)	5.80 (1.61)	6.05 (1.65)	5.80 (1.58)
T-Stat	−21.54^***^	−40.94^***^	−9.25^***^	−13.57^***^	−12.99^***^	−13.88^***^

The M = mean; SD = standard deviation; T-Stat are paired-samples *t* tests comparing pre- and post-intervention scores; ^***^p < 0.001. Higher scores indicate greater risk of AI addiction or stronger impact on the corresponding life domain.

Significant pre-post differences were also observed across all five domains of real-life functioning. Scores decreased in learning and work efficiency (M = 9.57 vs. 6.73), social interaction (M = 7.63 vs. 5.27), physical health (M = 8.20 vs. 5.80), hobbies and personal motivation (M = 8.45 vs. 6.05), and emotional wellbeing (M = 8.27 vs. 5.80).

## 5 Discussion

### 5.1 AI addiction risk and consequences

AI addiction exerts broad negative effects across multiple domains of university students' daily functioning, with severity generally increasing alongside addiction levels. Among the domains examined, learning and work efficiency were most strongly affected, followed by physical health, hobbies, and emotional wellbeing. Real-world social interactions were less consistently impacted, indicating that while AI dependence can disrupt daily life, its effects are domain specific.

Several potential factors may contribute to these patterns. First, the strong impact on learning and work efficiency suggests that habitual reliance on AI tools for task completion may foster dependence. Second, variability in social and emotional outcomes implies that limited offline support or unmet emotional needs can drive individuals to seek companionship or validation via AI interactions. Third, reductions in physical health and engagement with hobbies suggest that immersive AI experiences can dominate routines, further reinforcing excessive use.

### 5.2 Intervention effect evaluation

The six-month structured exercise intervention demonstrated apparent reductions in AI addiction risk among participants initially identified as at-risk. The improvements observed might imply that engaging in regular, appropriately designed physical activity can serve as an effective strategy for mitigating compulsive AI use. Participants reported positive perceptions of the intervention, reflecting both its acceptability and perceived usefulness.

Individual differences in outcomes highlight the moderating role of baseline factors such as initial addiction severity, physical fitness, and personal interest in exercise. These differences underscore the importance of tailoring interventions to participants' characteristics rather than applying uniform protocols. Gradual progression in exercise intensity, combined with a diverse selection of activities, may enhance both psychological and physiological responses while maintaining engagement.

Structured and personalized exercise programs appear to be an effective approach for reducing AI-related maladaptive behaviors among university students. Pre-intervention assessment of physical and mental status, addiction severity, and exercise preferences are recommended to guide the development of personalized programs. Continuous monitoring and feedback can further support adherence and optimize outcomes, promoting sustained reductions in AI dependence.

### 5.3 Limitations

Several limitations should be acknowledged.

First, the survey component adopted a cross-sectional design, which limits causal inference regarding the associations between problematic AI use and real-life functioning. Although an exercise-based intervention was subsequently conducted, the correlational findings from the cross-sectional phase do not establish directionality and alternative explanations remain possible. Longitudinal and experimental studies are needed to strengthen conclusions.

Second, all variables were assessed via self-report, which may introduce recall bias, social desirability effects, and common method variance. Future research should incorporate objective behavioral indicators or multi-method assessments to strengthen measurement validity.

Finally, the instrument assessing problematic AI use was developed with reference to established measures of Internet-related addiction (e.g., IAT and CIAS-R) and was applied in a non-clinical sample. The results therefore should be interpreted as reflecting addiction-like tendencies rather than clinically confirmed conditions. Further psychometric evaluation is required before stronger diagnostic claims can be drawn.

Overall, the present findings should be interpreted as exploratory and preliminary, providing a foundation for more rigorous future investigation.

## 6 Conclusion

This study examines potentially addictive AI use among Chinese university students and its associations with perceived negative impacts on real-life functioning. The findings indicate that difficulty in usage control, emotional dependence, cognitive offloading, and social dependence are key characteristics of maladaptive AI patterns. These dimensions are broadly consistent with prior research on problematic Internet use, social media addiction, and emerging studies on problematic use of conversational and generative AI systems, including ChatGPT-related use patterns. These features indicate that AI addiction extends beyond traditional Internet addiction, reflecting unique interaction patterns shaped by real-time adaptivity, anthropomorphic feedback, and personalized reinforcement algorithms.

Approximately 38% of respondents exhibited at least mild A-related maladaptive use, with negative impacts across academic performance, social interaction, physical health, personal interests, and emotional wellbeing.

A six-month structured exercise intervention for at-risk students yielded substantial improvements in this uncontrolled pre-post design, suggesting that physical activity may hold potential as a non-pharmacological strategy. Among 30 at-risk participants, 83.3% returned to normal usage levels, and mean AI addiction scores decreased from 48.87 to 34.50. Significant improvements were observed across all five domains of real-life functioning, with large effect sizes.

These findings are consistent with conceptualizing AI-related maladaptive use as a multidimensional pattern that shares features with traditional Internet addiction while also reflecting AI-specific interaction contexts. They provide preliminary evidence regarding the association between exercise-based intervention and reduced AI addiction risk levels in educational settings.

Taken together, these results underscore the necessity of recognizing AI addiction as an emerging behavioral issue that requires independent theoretical development and targeted intervention frameworks. The study's added value lies in integrating AI-specific interaction contexts with established behavioral addiction frameworks and examining their associations with perceived functional outcomes in a non-clinical student sample.

Future research should employ longitudinal and experimental designs, incorporate objective behavioral indicators, and further refine measurement tools to better capture heterogeneous AI interaction patterns. More rigorous intervention studies are also needed before firm conclusions can be drawn regarding the effectiveness of physical activity in reducing problematic AI use. Coordinated efforts across families, educational institutions, and communities will be essential for establishing sustainable support systems that promote healthy and balanced engagement with AI technologies in contemporary digital environments.

## Data Availability

The original contributions presented in the study are included in the article/Supplementary material, further inquiries can be directed to the corresponding author/s.

## References

[B1] Al-Obaydi L. Pikhart M. (2025). Artificial Intelligence Addiction: Exploring the Emerging Phenomenon of Addiction in the AI Age. Ai and Society. Cham: Springer Nature Switzerland AG. doi: 10.1007/s00146-025-02535-z

[B2] Brand M. Wegmann E. Stark R. Müller A. Wölfling K. Robbins T. . (2019). The interaction of person-affect-cognition-execution (I-PACE) model for addictive behaviors: update, generalization to addictive behaviors beyond internet-use disorders, and specification of the process character of addictive behaviors. Neurosci. Biobehav. Rev. 104, 1–10. doi: 10.1016/j.neubiorev.2019.06.032 31247240

[B3] Brown R. Abrantes A. Read J. Marcus B. Jakicic J. Strong D. . (2009). Aerobic exercise for alcohol recovery rationale, program description, and preliminary findings. Behav. Modif. 33, 220–249. doi: 10.1177/0145445508329112 19091721 PMC2829243

[B4] Chen H. Dong G. Li K. (2023). Overview on brain function enhancement of internet addicts through exercise intervention: based on reward-execution-decision cycle. Front. Psychiatry 14:1094583. doi: 10.3389/fpsyt.2023.1094583 36816421 PMC9933907

[B5] Davis R. (2001). A cognitive-behavioral model of pathological internet use. Comput. Human Behav. 17, 187–195. doi: 10.1016/S0747-5632(00)00041-8

[B6] Duong C. Dao T. Vu T. Ngo T. Tran Q. (2024). Compulsive ChatGPT usage, anxiety, burnout, and sleep disturbance: a serial mediation model based on stimulus-organism-response perspective. Acta Psychol. 251:104622. doi: 10.1016/j.actpsy.2024.104622 39647449

[B7] Hu B. Mao Y. Kim K. (2023). How social anxiety leads to problematic use of conversational AI: the roles of loneliness, rumination, and mind perception. Comput. Human Behav. 145:107760. doi: 10.1016/j.chb.2023.107760

[B8] Hu G.D. Zhang J. (2016). Research on effects of sports correcting adolescents with internet addiction disorder and its mechanism form the perspective of human instinct. China Sport Sci. Technol. 52, 68–77. doi: 10.16470/j.csst.201601010

[B9] Idrees A. Kraft R. Mutter A. Baumeister H. Reichert M. Pryss R. (2024). Persuasive technologies design for mental and behavioral health platforms: a scoping literature review. PLoS Digital Health 3:e0000498. doi: 10.1371/journal.pdig.0000498 38753889 PMC11098517

[B10] Kalivas P. Volkow N. (2005). The neural basis of addiction: a pathology of motivation and choice. Am. J. Psychiatry 162, 1403–1413. doi: 10.1176/appi.ajp.162.8.1403 16055761

[B11] Kuss D. Griffiths M. (2017). Social networking sites and addiction: ten lessons learned. Int. J. Environ. Res. Public Health. 14:311. doi: 10.3390/ijerph14030311 28304359 PMC5369147

[B12] Lai C. Mak K. Watanabe H. Ang R. Pang J. Ho R. (2013). Psychometric properties of the internet addiction test in Chinese adolescents. J. Pediatr. Psychol. 38, 794–807. doi: 10.1093/jpepsy/jst022 23671059

[B13] Mak K. K. Lai C. M. Ko C. H. Chou C. Kim D. I. Watanabe H. . (2014). Psychometric properties of the revised chen internet addiction scale (CIAS-R) in Chinese adolescents. J. Abnorm. Child Psychol. 42, 1237–1245. doi: 10.1007/s10802-014-9851-3 24585392

[B14] Moon S. Hwang J. Kim J. Shin A. Bae S. Kim J. (2018). Psychometric properties of the internet addiction test: a systematic review and meta-analysis. Cyberpsychol. Behav. Soc. Network. 21, 473–484. doi: 10.1089/cyber.2018.0154 30110200

[B15] Nie M. (2025). Algorithmic addiction by design: big tech's leverage of dark patterns to maintain market dominance and its challenge for content moderation. arXiv:2505.00054. Available online at: https://ui.adsabs.harvard.edu/abs/2025arXiv250500054N

[B16] Prochaska J. Diclemente C. Norcross J. (1992). In search of how people change - applications to addictive behaviors. Am. Psychol. 47, 1102–1114. doi: 10.1037/0003-066X.47.9.1102 1329589

[B17] Sparrow B. Liu J. Wegner D. (2011). Google effects on memory: cognitive consequences of having information at our fingertips. Science 333, 776–778. doi: 10.1126/science.1207745 21764755

[B18] Van Rooij A. Prause N. (2014). A critical review of “internet addiction” criteria with suggestions for the future. J. Behav. Addict. 3, 203–213. doi: 10.1556/JBA.3.2014.4.1 25592305 PMC4291825

[B19] Wilmer H. Sherman L. Chein J. (2017). Smartphones and cognition: a review of research exploring the links between mobile technology habits and cognitive functioning. Front. Psychol. 8:605. doi: 10.3389/fpsyg.2017.00605 28487665 PMC5403814

[B20] Yankouskaya A. Liebherr M. A. R. (2025). Can chatgpt be addictive? a call to examine the shift from support to dependence in AI conversational large language models. Hum. Cent. Intell. Syst. 5, 77–89. doi: 10.1007/s44230-025-00090-w

[B21] Young K. (2004). Internet addiction - a new clinical phenomenon and its consequences. Am. Behav. Sci. 48, 402–415. doi: 10.1177/0002764204270278

[B22] Young K. S. (1998). Internet addiction: the emergence of a new clinical disorder. CyberPsychol. Behav. 1, 237–244. doi: 10.1089/cpb.1998.1.237

[B23] Zhou T. Zhang C. (2024). Examining generative AI user addiction from a C-A-C perspective. Technol. Soc. 78:102653. doi: 10.1016/j.techsoc.2024.102653

