# Supplementary material for: Artificial intelligence addiction among university students in China: risk stratification, consequences, and exercise-based intervention
Source: Front Psychol. 2026 Jun 25;17:1779639. doi: 10.3389/fpsyg.2026.1779639 (PMC13346038; doi:10.3389/fpsyg.2026.1779639)
Supplement: Supplementary file 1 [file Table_1.docx]

**AI Tools and Services Usage Survey**

**1. Basic Information**

**Q1: Your age:**

| **Option** | **Count** | **Percentage** |
| --- | --- | --- |
| Under 18 | 6 | 2.49% |
| 18–24 years old | 232 | 96.27% |
| 24–30 years old | 3 | 1.24% |
| Over 30 years old | 0 | 0% |

**Q2: Your gender:**

| **Option** | **Count** | **Percentage** |
| --- | --- | --- |
| Male | 121 | 50.21% |
| Female | 120 | 49.79% |

**Q3: Your grade :**

| **Option** | **Count** | **Percentage** |
| --- | --- | --- |
| Freshman | 90 | 37.97% |
| Sophomore | 101 | 42.62% |
| Junior | 35 | 14.77% |
| Senior or above | 11 | 4.64% |

**2. AI Usage Section**

Note: The following questions assess your usage of AI-related tools or services (e.g., short video platforms with recommendation systems, social media, AI-generated content tools, etc.). Please select the option that best matches your actual feelings and behavior frequency.

**Q4: Behavioral dependence (Matrix scale question)**

| **Question** | **Never** | **Occasionally** | **Sometimes** | **Often** | **Always** | **Average** |
| --- | --- | --- | --- | --- | --- | --- |
| I often spend more time using AI tools/services than planned. | 20 (8.3%) | 47 (19.5%) | 67 (27.8%) | 78 (32.37%) | 29 (12.03%) | 3.2 |
| Even knowing that using AI tools/services has affected my normal study/work, I still can't control my usage. | 37 (15.35%) | 39 (16.18%) | 78 (32.37%) | 55 (22.82%) | 32 (13.28%) | 3.02 |
| I have tried to limit my use of AI tools/services but failed. | 61 (25.31%) | 50 (20.75%) | 69 (28.63%) | 40 (16.6%) | 21 (8.71%) | 2.63 |
| I neglect other important things because of using AI tools/services. | 66 (27.39%) | 51 (21.16%) | 60 (24.9%) | 47 (19.5%) | 17 (7.05%) | 2.58 |
| **Total** | **184 (19.09%)** | **187 (19.4%)** | **274 (28.42%)** | **220 (22.82%)** | **99 (10.27%)** | **2.86** |

**Q5: Emotional dependence (Matrix scale question)**

| **Question** | **Never** | **Occasionally** | **Sometimes** | **Often** | **Always** | **Average** |
| --- | --- | --- | --- | --- | --- | --- |
| When feeling low, I use AI tools/services to relieve my mood. | 79 (32.78%) | 40 (16.6%) | 50 (20.75%) | 48 (19.92%) | 24 (9.96%) | 2.58 |
| I look forward to the "instant feedback" feeling from AI tools/services. | 61 (25.31%) | 52 (21.58%) | 46 (19.09%) | 50 (20.75%) | 32 (13.28%) | 2.75 |
| When not using AI tools/services, I feel anxious or irritable. | 75 (31.12%) | 53 (21.99%) | 51 (21.16%) | 34 (14.11%) | 28 (11.62%) | 2.53 |
| I need to use AI tools/services for longer periods to feel satisfied. | 94 (39%) | 39 (16.18%) | 53 (21.99%) | 38 (15.77%) | 17 (7.05%) | 2.36 |
| **Total** | **309 (32.05%)** | **184 (19.09%)** | **200 (20.75%)** | **170 (17.63%)** | **101 (10.48%)** | **2.55** |

**Q6: Cognitive dependence (Matrix scale question)**

| **Question** | **Never** | **Occasionally** | **Sometimes** | **Often** | **Always** | **Average** |
| --- | --- | --- | --- | --- | --- | --- |
| After using AI tools, I rarely think about problems myself. | 24 (9.96%) | 60 (24.9%) | 63 (26.14%) | 63 (26.14%) | 31 (12.86%) | 3.07 |
| I rely more and more on AI to help me make decisions or judgments. | 40 (16.6%) | 55 (22.82%) | 68 (28.22%) | 49 (20.33%) | 29 (12.03%) | 2.88 |
| Without AI tools, I feel less efficient. | 21 (8.71%) | 37 (15.35%) | 75 (31.12%) | 77 (31.95%) | 31 (12.86%) | 3.25 |
| I tend to use AI-generated content directly rather than creating it myself. | 37 (15.35%) | 58 (24.07%) | 72 (29.88%) | 48 (19.92%) | 26 (10.79%) | 2.87 |
| **Total** | **122 (12.66%)** | **210 (21.78%)** | **278 (28.84%)** | **237 (24.59%)** | **117 (12.14%)** | **3.02** |

**Q7: Social dependence (Matrix scale question)**

| **Question** | **Never** | **Occasionally** | **Sometimes** | **Often** | **Always** | **Average** |
| --- | --- | --- | --- | --- | --- | --- |
| I prefer chatting or interacting with AI rather than with real people. | 99 (41.08%) | 46 (19.09%) | 43 (17.84%) | 38 (15.77%) | 15 (6.22%) | 2.27 |
| I feel that AI understands me better than real friends. | 115 (47.72%) | 31 (12.86%) | 41 (17.01%) | 33 (13.69%) | 21 (8.71%) | 2.23 |
| I rely on AI for companionship or responses to alleviate loneliness. | 95 (39.42%) | 44 (18.26%) | 45 (18.67%) | 37 (15.35%) | 20 (8.3%) | 2.35 |
| I find AI more reliable than real people. | 107 (44.4%) | 35 (14.52%) | 47 (19.5%) | 35 (14.52%) | 17 (7.05%) | 2.25 |
| **Total** | **416 (43.15%)** | **156 (16.18%)** | **176 (18.26%)** | **143 (14.83%)** | **73 (7.57%)** | **2.27** |

**3. Real-life Impact Module**

Note: This section further assesses the impact of AI-related tools/services on your real-life functioning. Please select the option that best matches your experience over the past month.

**Q8: Learning/work efficiency (Matrix scale question)**

| **Question** | **Strongly disagree** | **Disagree** | **Neutral** | **Agree** | **Strongly agree** | **Average** |
| --- | --- | --- | --- | --- | --- | --- |
| Relying on AI tools (e.g., generating reports/solutions) has led to a decline in my actual work ability (e.g., independent writing/calculation speed). | 22 (9.13%) | 44 (18.26%) | 77 (31.95%) | 69 (28.63%) | 29 (12.03%) | 3.16 |
| When faced with urgent tasks that must be completed independently, I feel strong anxiety. | 30 (12.45%) | 29 (12.03%) | 75 (31.12%) | 79 (32.78%) | 28 (11.62%) | 3.19 |
| Due to over-reliance on AI-generated content, I performed poorly in important assessments where AI was not allowed. | 35 (14.52%) | 65 (26.97%) | 73 (30.29%) | 48 (19.92%) | 20 (8.3%) | 2.8 |
| **Total** | **87 (12.03%)** | **138 (19.09%)** | **225 (31.12%)** | **196 (27.11%)** | **77 (10.65%)** | **3.05** |

**Q9: Real-life social interaction (Matrix scale question)**

| **Question** | **Strongly disagree** | **Disagree** | **Neutral** | **Agree** | **Strongly agree** | **Average** |
| --- | --- | --- | --- | --- | --- | --- |
| I prefer using AI for communication rather than accepting offline social invitations (e.g., friend gatherings). | 82 (34.02%) | 55 (22.82%) | 51 (21.16%) | 32 (13.28%) | 21 (8.71%) | 2.4 |
| When communicating with real people, I find it less relaxed or appealing than with AI. | 91 (37.76%) | 48 (19.92%) | 44 (18.26%) | 38 (15.77%) | 20 (8.3%) | 2.37 |
| I find myself spending less time and effort planning or participating in offline social activities. | 77 (31.95%) | 52 (21.58%) | 53 (21.99%) | 41 (17.01%) | 18 (7.47%) | 2.46 |
| **Total** | **250 (34.58%)** | **155 (21.44%)** | **148 (20.47%)** | **111 (15.35%)** | **59 (8.16%)** | **2.41** |

**Q10: Physical health (Matrix scale question)**

| **Question** | **Strongly disagree** | **Disagree** | **Neutral** | **Agree** | **Strongly agree** | **Average** |
| --- | --- | --- | --- | --- | --- | --- |
| After prolonged use of AI tools/services, I experience blurred vision or dizziness. | 55 (22.82%) | 53 (21.99%) | 64 (26.56%) | 51 (21.16%) | 18 (7.47%) | 2.68 |
| Due to late-night use of AI tools/services, my sleep duration has decreased. | 64 (26.56%) | 55 (22.82%) | 63 (26.14%) | 39 (16.18%) | 20 (8.3%) | 2.57 |
| Frequent use of AI tools/services has increased the frequency of neck or shoulder pain. | 64 (26.56%) | 51 (21.16%) | 60 (24.9%) | 45 (18.67%) | 21 (8.71%) | 2.62 |
| **Total** | **183 (25.31%)** | **159 (21.99%)** | **187 (25.86%)** | **135 (18.67%)** | **59 (8.16%)** | **2.62** |

**Q11: Hobbies (Matrix scale question)**

| **Question** | **Strongly disagree** | **Disagree** | **Neutral** | **Agree** | **Strongly agree** | **Average** |
| --- | --- | --- | --- | --- | --- | --- |
| I have shifted more time from non-AI activities (e.g., painting/reading/exercise) to using AI tools/services. | 55 (22.82%) | 62 (25.73%) | 59 (24.48%) | 45 (18.67%) | 20 (8.3%) | 2.64 |
| Spending more time on AI tools/services has reduced my sense of fulfillment in previously sustained non-AI activities. | 59 (24.48%) | 66 (27.39%) | 57 (23.65%) | 42 (17.43%) | 17 (7.05%) | 2.55 |
| **Total** | **114 (23.65%)** | **128 (26.56%)** | **116 (24.07%)** | **87 (18.05%)** | **37 (7.68%)** | **2.6** |

**Q12: Emotional state (Matrix scale question)**

| **Question** | **Strongly disagree** | **Disagree** | **Neutral** | **Agree** | **Strongly agree** | **Average** |
| --- | --- | --- | --- | --- | --- | --- |
| When AI tools/services are unavailable, my emotions become unstable (e.g., irritable, low). | 53 (21.99%) | 65 (26.97%) | 56 (23.24%) | 45 (18.67%) | 22 (9.13%) | 2.66 |
| Natural pleasures in real life feel numb to me; only AI can satisfy me. | 79 (32.78%) | 65 (26.97%) | 46 (19.09%) | 31 (12.86%) | 20 (8.3%) | 2.37 |
| If I cannot use AI tools/services for a long time, I feel uneasy or uncomfortable. | 57 (23.65%) | 63 (26.14%) | 54 (22.41%) | 46 (19.09%) | 21 (8.71%) | 2.63 |
| **Total** | **189 (26.14%)** | **193 (26.69%)** | **156 (21.58%)** | **122 (16.87%)** | **63 (8.71%)** | **2.55** |

**4. Physical Exercise Module**

Note: The following questions aim to understand your physical exercise habits, interests, and views on the relationship between exercise and mental health. Please select the option that best matches your actual situation.

**Q13: How often do you currently engage in physical exercise?**

| **Option** | **Count** | **Percentage** |
| --- | --- | --- |
| Never | 22 | 9.13% |
| 1–2 times per month | 73 | 30.29% |
| 1–2 times per week | 71 | 29.46% |
| 3–4 times per week | 38 | 15.77% |
| 5 or more times per week | 37 | 15.35% |

**Q14: How long is each exercise session approximately?**

| **Option** | **Count** | **Percentage** |
| --- | --- | --- |
| <10 minutes | 32 | 14.61% |
| 10–30 minutes | 103 | 47.03% |
| 30–60 minutes | 51 | 23.29% |
| 1–2 hours | 33 | 15.07% |

**Q15: What types of exercise do you usually choose? (Multiple choice)**

| **Option** | **Count** | **Percentage** |
| --- | --- | --- |
| Ball sports (basketball, football, badminton, etc.) | 119 | 54.34% |
| Running/Walking | 145 | 66.21% |
| Fitness/Strength training | 63 | 28.77% |
| Yoga/Pilates | 39 | 17.81% |
| Dance/Street dance | 27 | 12.33% |
| Other (please specify) | 14 | 6.39% |

**Q16: What are your main reasons for engaging in physical exercise? (Multiple choice)**

| **Option** | **Count** | **Percentage** |
| --- | --- | --- |
| Maintaining health | 152 | 69.41% |
| Relieving stress | 121 | 55.25% |
| Social needs (exercising with friends) | 76 | 34.7% |
| Pursuing a sense of achievement (e.g., skill improvement) | 68 | 31.05% |
| Weight loss or body shaping | 91 | 41.55% |
| Other (please specify) | 9 | 4.11% |

**Q17: Are you interested in trying new sports activities?**

| **Option** | **Count** | **Percentage** |
| --- | --- | --- |
| Yes | 185 | 76.76% |
| No | 56 | 23.24% |

**Q18: What do you think are the main factors hindering your persistence in exercise? (Multiple choice)**

| **Option** | **Count** | **Percentage** |
| --- | --- | --- |
| A. Lack of time | 144 | 59.75% |
| B. Lack of interest | 122 | 50.62% |
| C. Lack of companions | 139 | 57.68% |
| D. Venue or equipment limitations | 115 | 47.72% |
| E. Other (please specify) | 10 | 4.15% |

**Q19: Which of the following methods do you think would best motivate you to persist in exercise? (Multiple choice)**

| **Option** | **Count** | **Percentage** |
| --- | --- | --- |
| A. Regular exercise reminders | 114 | 47.3% |
| B. Group check-in supervision | 120 | 49.79% |
| C. Personalized exercise recommendations | 162 | 67.22% |
| D. Courses combining mental health | 100 | 41.49% |
| E. Other (please specify) | 9 | 3.73% |

**5. Intervention Strategies Module**

Note: This section assesses the relationship between exercise and AI addiction. Please select the option that best matches your experience over the past month.

**Q20: The intervention effect of exercise (Matrix scale)**

| **Question** | **Strongly disagree** | **Disagree** | **Neutral** | **Agree** | **Strongly agree** | **Average** |
| --- | --- | --- | --- | --- | --- | --- |
| After exercise, my daily routine becomes more regular. | 15 (6.22%) | 22 (9.13%) | 47 (19.5%) | 111 (46.06%) | 46 (19.09%) | 3.63 |
| After exercise, my mental state improves. | 10 (4.15%) | 20 (8.3%) | 53 (21.99%) | 102 (42.32%) | 56 (23.24%) | 3.72 |
| Exercise helps me reduce the time spent on AI tools/services. | 23 (9.54%) | 38 (15.77%) | 80 (33.2%) | 65 (26.97%) | 35 (14.52%) | 3.21 |
| The happiness and satisfaction from exercise reduce my need to seek emotional comfort through AI. | 26 (10.79%) | 28 (11.62%) | 81 (33.61%) | 72 (29.88%) | 34 (14.11%) | 3.25 |
| Consistent exercise improves my self-control, helping me reduce excessive use of AI tools/services. | 19 (7.88%) | 40 (16.6%) | 69 (28.63%) | 81 (33.61%) | 32 (13.28%) | 3.28 |
| After long-term exercise, my anxiety when unable to use AI tools/services decreases. | 21 (8.71%) | 32 (13.28%) | 81 (33.61%) | 72 (29.88%) | 35 (14.52%) | 3.28 |
| Exercise promotes my face-to-face social interactions, reducing my dependence on virtual socializing. | 17 (7.05%) | 35 (14.52%) | 67 (27.8%) | 78 (32.37%) | 44 (18.26%) | 3.4 |
| When feeling bored or anxious, I prioritize exercise over AI tools/services. | 12 (4.98%) | 39 (16.18%) | 72 (29.88%) | 76 (31.54%) | 42 (17.43%) | 3.4 |
| Long-term exercise makes me realize that many needs previously satisfied by AI tools/services can be met through exercise. | 22 (9.13%) | 38 (15.77%) | 71 (29.46%) | 81 (33.61%) | 29 (12.03%) | 3.24 |
| If there were a sports app providing instant feedback similar to AI (e.g., achievement badges, social interaction), I would be more willing to exercise. | 17 (7.05%) | 27 (11.2%) | 73 (30.29%) | 84 (34.85%) | 40 (16.6%) | 3.43 |
| **Total** | **182 (7.55%)** | **319 (13.24%)** | **694 (28.8%)** | **822 (34.11%)** | **393 (16.31%)** | **3.38** |

**Q21: In what aspects do you think exercise regulates addictive behavior? (Multiple choice)**

| **Option** | **Count** | **Percentage** |
| --- | --- | --- |
| A. Emotional regulation | 181 | 75.1% |
| B. Time management | 152 | 63.07% |
| C. Social interaction | 125 | 51.87% |
| D. Sense of achievement enhancement | 120 | 49.79% |
| Other (please specify) | 1 | 0.41% |

**Q22: In what aspects do you think exercise improves mental health? (Multiple choice)**

| **Option** | **Count** | **Percentage** |
| --- | --- | --- |
| A. Relieving anxiety | 184 | 76.35% |
| B. Reducing depression | 160 | 66.39% |
| C. Improving concentration | 157 | 65.15% |
| D. Enhancing self-control | 157 | 65.15% |
| E. Other (please specify) | 3 | 1.24% |
